# Risk prediction of kidney function in long-term kidney transplant recipients

**DOI:** 10.3389/fmed.2025.1469363

**Published:** 2025-03-20

**Authors:** Krzysztof Batko, Anna Sączek, Małgorzata Banaszkiewicz, Jolanta Małyszko, Ewa Koc-Żórawska, Marcin Żórawski, Karolina Niezabitowska, Katarzyna Siek, Alina Bętkowska-Prokop, Andrzej Kraśniak, Marcin Krzanowski, Katarzyna Krzanowska

**Affiliations:** ^1^Department of Dermatology, Jagiellonian University Medical College, Kraków, Poland; ^2^Department of Nephrology and Transplantology, Jagiellonian University Medical College, Cracow, Poland; ^3^Department of Nephrology, Dialysis and Internal Medicine, Warsaw Medical University, Warsaw, Poland; ^4^2nd Department of Nephrology, Hypertension and Internal Medicine with Dialysis Unit, Medical University of Bialystok, Bialystok, Poland; ^5^Department of Cardiology, Lipidology and Internal Medicine with Cardiac Intensive Care Unit, Medical University of Bialystok, Bialystok, Poland; ^6^The Academy of Applied Medical and Social Sciences, Elbląg, Poland

**Keywords:** kidney, ELA, APLN, risk prediction, machine learning, transplantation

## Abstract

**Background:**

Limited tools exist for predicting kidney function in long-term kidney transplant recipients (KTRs). Elabela (ELA), apelin (APLN), and the APJ receptor constitute an axis that regulates vascular and cardiac physiology in opposition to the renin-angiotensin-aldosterone system.

**Methods:**

Longitudinal, observational cohort of 102 KTRs who maintained graft function for at least 24 months, with no acute rejection history or active infection upon presentation. Serum APLN, ELA, fibroblast growth factor 23 (FGF-23) and α Klotho were tested using enzyme-linked immunoassay and compared with a control group of 32 healthy controls (HCs).

**Results:**

When comparing with HCs, higher serum FGF-23, ELA and APLN, but lower ɑ Klotho concentrations were observed in long-term KTRs. Most KTRs had stable trajectories of renal function. Mean estimated glomerular filtration (eGFR) over 2-year follow-up was associated with significantly lower odds of graft loss (OR 0.04, 95% CI 0.01–0.15; *p* < 0.001). Baseline renal function was significantly correlated with mineral–bone markers (log[FGF-23]: *r* = −0.24, *p* = 0.02; log[α-Klotho]: *r* = 0.34, *p* < 0.001) but showed no significant association with aplnergic peptides (APLN: *r* = −0.07, *p* = 0.51; ELA: *r* = 0.17, *p* = 0.10). Univariable random forest regression indicated that baseline eGFR alone explained 87% of the variance in future 2-year eGFR, suggesting its overarching importance in late-term predictions. Incorporating both simple clinical characteristics and candidate serum biomarkers into a model predicting last available eGFR allowed for moderate predictive performance. In univariable Cox Proportion Hazard models, lower log(α-Klotho) (HR 0.26, 95% CI 0.12–0.58; *p* = 0.001) and higher log(FGF-23) (HR 2.14, 95% CI 1.49–3.09; *p* < 0.001) were significant predictors of death-censored allograft loss.

**Conclusion:**

Both aplnergic and mineral-bone peptides appear as relevant candidate markers for future studies investigating their predictive performance regarding renal allograft outcomes.

## 1 Introduction

Chronic kidney disease (CKD) is an irreversible disorder with a slowly progressive course that is tied to excess morbidity. Studies estimate its global prevalence at over 10% within the general population, with non-linear growth and even higher rates among the elderly (1). Cardiovascular (CV) disease remains one of the leading causes of death across the whole spectrum of CKD. CV-related mortality is starkly elevated in end-stage CKD, especially in subjects requiring renal replacement therapy (RRT), but also remains significantly elevated after kidney transplant (KTx) (2).

Apelin (APLN) and Elabela (ELA; toddler or apela) are two endogenous ligands of the APJ, a G protein coupled receptor tied to several intracellular transduction pathways affecting adenylyl cyclase activity, ion gradients (including calcium shift) and nitric oxide synthesis (3, 4). APJ shows widespread expression in multiple tissues, including cardiac, vascular and renal organs (1, 5, 6). The APJ receptor shows modest homology and comparable tissue distribution to angiotensin receptor type 1, which has led to speculation of its physiological role as a counterbalance to the renin-angiotensin-aldosteron (RAAS) axis, alterations of which result in accelerated atherosclerosis and various organ disorders (7–10).

APLN and ELA are 77- and 54-amino acid preproproteins cleaved by tissue proteases into shorter peptides, which are secretable (11). While the C-terminal APLN sequence facilitates receptor binding, the N-terminal further interacts with the APJ receptor, which may explain variability in tissue affinity of specific isoforms (1, 12). Most immunological assays target the C-terminal fragment of ELA, which precludes differentiation of specific isoforms. Immunoassay remains the only robust method of testing ELA concentrations (13, 14). Contrary to prior hypotheses suspecting an ELA-specific receptor, ELA has been demonstrated to act as a ligand of the APJ receptor, with near selective expression within the vascular endothelium of the kidney (15–17).

In contrast to the early post-Ktx period, long-term kidney transplant recipients (KTRs) represent a distinct population that is particularly susceptible to CV and metabolic diseases (e.g., longer use of immunosuppresion and related toxicity). These comorbid disorders also represent a major risk factor and leasing cause of graft loss and/or non-renal death (18). Developing prognostic tools for these patients is of particular interest, due to the paucity of measures that are validated in this special population. Due to its strong link with CV disease, the recently discovered aplnergic axis represents an important regulator of tissue homeostasis and potential source of biomarkers.

While complex models derived based on large populations of KTRs have been previously published, the population of stable, long-term KTRs represent a unique group. This study was undertaken to explore the relationship between circulating concentrations of aplnergic and mineral-bone peptides with allograft function in long-term KTRs. The primary outcome of interest in this study was the prognostic relevance of aplnergic peptides regarding renal allograft function, particularly given the close integration of cardio-renal-vascular disease. We also aimed to describe and cross-examine different modeling approaches for outcomes of renal importance through a combination of traditional statistical methods and machine-learning techniques.

## 2 Materials and methods

### 2.1 Study design and population

This was a longitudinal, observational cohort study, which enrolled 102 consecutive patients under ambulatory care at the University Hospital in Kraków, which is the highest-grade reference center for the Malopolska region (~3,4 million inhabitants) in Poland. The enrollment process had an annual timeframe between September 1, 2016 and October 31, 2017. The recruitment pool was an outpatient population of 1182 KTRs presenting for control visits. Subjects were considered eligible only if they were long-term KTRs, which was defined as having maintained allograft function for at least 24 months. Furthermore, since episodes of acute rejection remain a major risk factor for chronic rejection (19), we excluded subjects in whom history of acute rejection was recorded (whether cellular, humoral or vascular). Exclusion criteria also comprised infection, both active (i.e., signs or symptoms of any acute infection at presentation) or chronic (based on serological evidence in routine work-up, e.g., viral hepatitis, HIV). Due to altered mineral-bone imbalance, individuals with prior parathyroidectomy status or a history of malignancy (excluding non-melanoma skin cancer), were not considered due to the presence of potential interfering factors.

### 2.2 Follow-up

Over time, electronic medical care records were screened manually by four physicians to identify visits at relatively equally spaced intervals, with a pre-defined range of 3–6 months apart. The total follow-up time for renal function measures was comparable for most patients, with a median (IQR) timeframe of 23.6 (22.8–24.4) months and range of 21.7–25.3 months. The occurrence of events of poor prognosis, such as allograft loss (otherwise referred to as “allograft dysfunction”; defined as permanent dialysis transfer or re-transplantation listing, ascertained irrespective of eGFR assessments due to potential time lag / missing data) or all-cause death was determined through in person or telephone contact with the patient, family and/or dialysis center. This process was carried out successively until December 30, 2023 (censoring date). Overall, 25 patients experienced allograft loss and 20 died. Of the latter, the majority were characterized as “death with functioning graft” (*N* = 13, 65%).

### 2.3 Model features

Clinical data, which included age, sex, body mass index (BMI), KTx characteristics (primary etiology of kidney disease, immunosuppressive treatment, history of delayed graft function) and co-morbidity (CV disease, hypertension, diabetes and dyslipidemia) were gathered. CV disease was defined as records of either atherosclerotic CV disease (i.e., a composite of coronary artery disease (CAD), prior myocardial infarction (MI) or prior stroke) or heart failure (HF). Nearly all patients were treated with triple immunosuppressive therapy (glucocorticoids, mycophenolate mofetil and a calcineurin inhibitor) and none had history of induction pre-treatment. Glomerulonephritis, autosomal dominant polycystic kidney disease and reflux nephritis were the most common primary causes of KTx.

### 2.4 Biochemical testing

Peripheral venous cannulation was performed at presentation on the morning following an overnight fast into EDTA tubes, which were subsequently frozen at −70 C and stored until analysis. Standard biochemistry assays were performed on either of three automatic analyzers: Hitachi 917 (Hitachi, Japan); Modular P (Roche Diagnostics, Germany); Sysmex XE 2100 (Sysmex, Japan). For non-routine biochemical assays, testing was performed in batch, using commercially available immunoenzymatic kits according to manufacturer instructions. A healthy control group of 32 volunteers (16 female; 16 male), aged between 29 and 74 years (mean 50 years) was recruited using convenience sampling among willing physicians and medical personnel. A more detailed description of this control group is available elsewhere (20).

Serum APLN was measured using Human APLN ELISA Kit (EIAab Science, Wuhan, China), with a detectable level of 62.5–4,000 pg/mL, sensitivity of 30 pg/mL, and intra- and inter-assay precision of <7 and <9%, respectively. Serum ELA was measured using Cat. No. S-1508 ELABELA (Peninsula Laboratories, San Carlos, CA, United States), with a detectable level of 0–100 ng/mL, and intra- and inter-assay precision ~10% and ~15% [as reported (21)], respectively. No cross-reactivity with APLN has been reported. Serum klotho was measured using Human Soluble α Klotho kit (IBL, Gunma, Japan), with a detectable level of 93.75–6,000 pg/mL, sensitivity of 6.15 pg/mL, and intra- and inter-assay precision of ~3% and ~ 7%, respectively. Serum FGF-23 was measured using Human FGF-23 Intact ELISA kit (Immunotopics, San Clemente, United States), with a detectable level of 31.25–2,000 pg/mL, sensitivity of 15 pg/mL, and intra- and inter-assay precision of ~7% and ~11%, respectively.

### 2.5 Statistical analysis

Analyses were performed in R v4.4.1 (R Core Team, 2024, R Foundation for Statistical Computing, Vienna, Austria). Nominal variables were summarized as counts and proportions (N, %). Variable distribution was assessed using density plots, the Shapiro Wilk test and skewness measures. Continuous variables were summarized as mean (standard deviation) or median (interquartile range), as deemed appropriate. Comparison across groups is performed using robust tests (*WRS2* package) for continuous variables. The measure of association for two dichotomous variables was assessed with x^2^ test or Fisher’s exact test. The *nephro* package was used to calculate estimated glomerular filtration (eGFR) according to the Chronic Kidney Disease-Epidemiology Collaboration (CKD-EPI) formula based on serum creatinine (22). Statistical tests were two-tailed and *p* < 0.05 was deemed statistically significant.

Prognostic models were constructed using ranger within the tidymodels framework, as a high-performance machine learning model, using the *ranger* package within the *tidymodels* package. Due to generally low rates of missing data per variable (<10%), we utilized imputation using bagging techniques. For our primary endpoint, although the median is often considered more robust against outliers, we observed a very high correlation between the median and the mean eGFR. Moreover, the distributional properties of the data supported using the mean. Therefore, we employed the mean eGFR over 2-year follow-up as the response variable in our predictor development models. This metric serves as an approximation of the “true” short-term renal function and is less sensitive to measurement variability and transient events that might impact an isolated measurement.

Conversely, to more reliably assess the validity of the developed models, we used the last available eGFR measurement from subsequent follow-up for final model construction. Although this approach introduces a variable time horizon for the outcome, it reduces the risk of overfitting compared to using the repeated measures used of the predictor development phase.

Tuning was performed with grid search for hyperparameter selection using five times repeated tenfold cross-validation. Permutation-based model breakdown techniques using the *vip* and *DALEX* package were utilized to analyze feature contribution to model prediction from a global and local perspective, respectively.

## 3 Results

### 3.1 Baseline clinical characteristics of kidney transplant recipients

This was a cohort of middle-aged adults, with mean (SD) age of 50.8 (14.5) years and male predominance (*N* = 72, 70.6%). Most patients were overweight, with mean (SD) body mass of 26.1 (4.68) kg/m^2^. Tacrolimus triple therapy regimens were most utilized (*N* = 68, 66.7%), followed by cyclosporine-based schemes (*N* = 22, 21.6%). The history of delayed graft function, defined as dialysis requirement within 7 days in the early Tx period, was documented for every fifth patient (*N* = 23, 22.5%). For mortality and kidney allograft loss, the median (IQR) follow-up time was 82.6 (42.1–85.2) months. Most patients retained adequate kidney function at baseline, with mean eGFR upon presentation at 58.4 (22.6) ml//min/1.73m^2^, respectively.

The majority of subjects were hypertensive (*N* = 92, 90.2%), with dyslipidemia and diabetes reported for 15 (14.7%) and 29 (28.4%) individuals, respectively. Nearly all diabetics were characterized with post-transplant diabetes. Manifest CV disease, defined as any form of atherosclerotic vascular disease, prior cardiovascular event history or concomitant heart failure, was observed in every fourth patient (*N* = 25, 24.5%). A comparison of clinical features according to kidney graft status at last available follow-up is shown in Table 1.

**Table 1:** Comparison of baseline clinical features and biochemical assessments according to allograft status at the end of follow-up.

	Allograft survival (*N* = 77)	Allograft dysfunction (*N* = 25)	*p* value
Age, years	52.03 (14.33)	47.12 (14.71)	0.23
Male sex, *N* (%)	52 (67.5%)	19 (79.2%)	0.24
BMI, kg/m^2^	25.81 (4.73)	26.91 (4.50)	0.20
Baseline eGFR, ml/min	64.38 (20.82)	40.09 (17.94)	<0.001
CKD stage, *N* (%)			<0.001
V-IV	2 (2.6%)	7 (28.0%)	
IIIB	14 (18.2%)	13 (52.0%)	
IIIA	19 (24.7%)	1 (4.0%)	
II	33 (42.9%)	3 (12.0%)	
I	9 (11.7%)	1 (4.0%)	
CVD, *N* (%)	16 (20.8%)	9 (36.0%)	0.12
Hypertension, *N* (%)	69 (89.6%)	23 (92.0%)	0.73
MAP, mm Hg	99.98 (11.93)	103.03 (13.86)	0.49
Dyslipidemia	10 (13.0%)	5 (20.8%)	0.13
Diabetes, *N* (%)	9 (11.7%)	6 (24.0%)	0.14
Fasting glucose, mmol/l	5.63 (1.39)	5.75 (0.90)	0.29
Hemoglobin, g/dL	13.75 (2.12)	13.92 (1.46)	0.93
FGF-23, pmol/L	0.96 (0.66–1.34)	1.33 (1.01–4.38)	0.57
α Klotho, pg/mL	646.60 (554.80–871.90)	452.70 (392.90–635.60)	<0.001
ELA, ng/mL	28.75 (25.72–38.26)	28.68 (23.21–37.40)	<0.001
APLN, pg/mL	1,241 (907.50–1,548)	1,173 (1,080–1,805)	0.66

Allograft dysfunction was defined as permanent dialysis transfer or kidney transplantation re-listing.

BP, blood pressure; BMI, body mass index; eGFR, estimated glomerular filtration; CVD, manifest cardiovascular disease; FGF-23, fibroblast growth factor type 23; MAP, mean arterial blood pressure.

**Figure 1:**
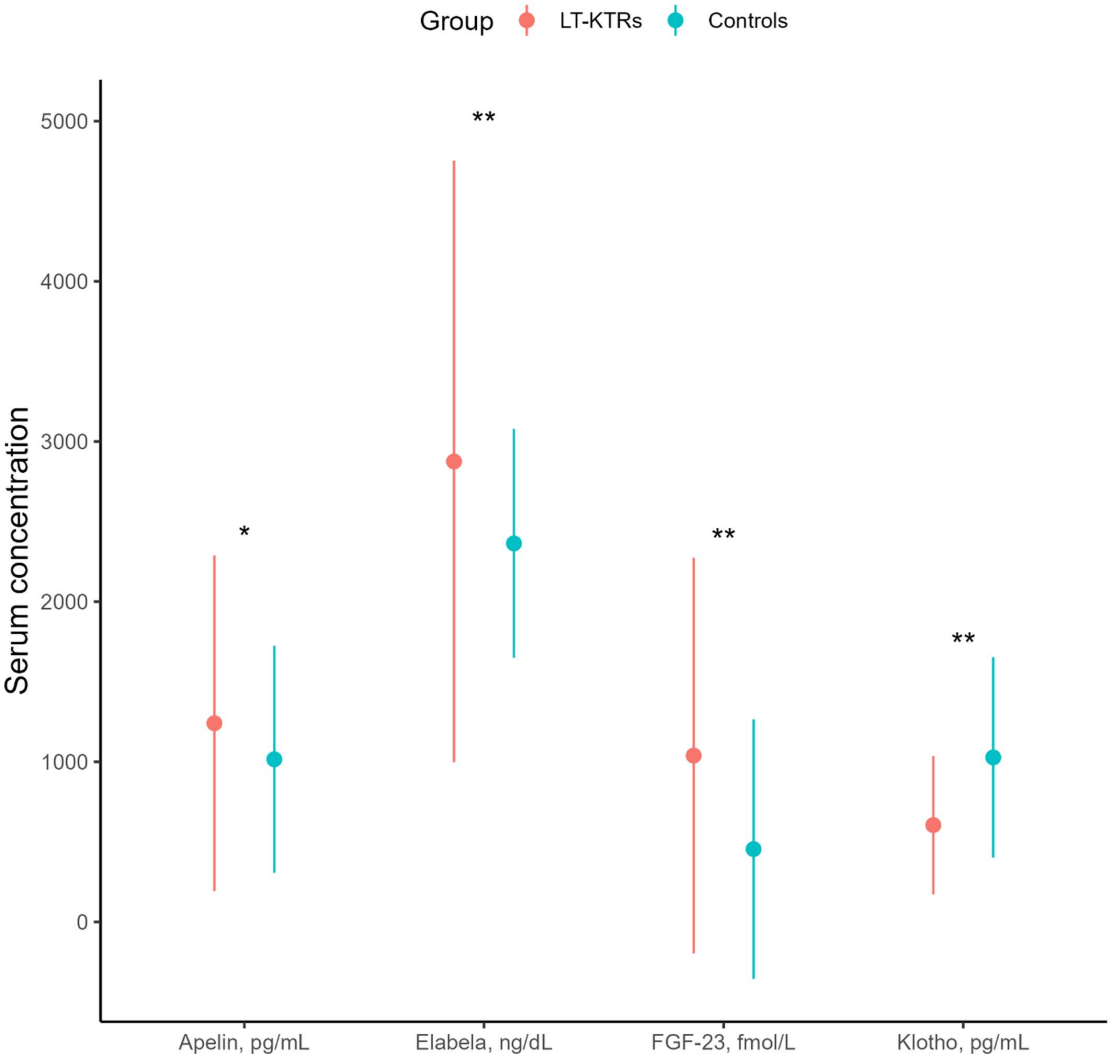
Differences in serum concentrations of APLN, ELA, fibroblast growth factor type 23 (FGF-23) and alpha klotho compared between long-term kidney transplant recipients (LT-KTRs) and healthy controls. Point range is based on median with 1.5 * IQR for whiskers. *, **p value ≤ 0.05 and 0.001.

When comparing with healthy controls, we observed higher median concentrations of serum FGF-23 (*p* = 0.001), ELA (*p* = 0.001) and APLN (*p* = 0.041) in KTRs, alongside significantly lower α-Klotho (*p* = 0.001) levels (Figure 1).

### 3.2 Relationships between kidney function, vascular and metabolic parameters and circulating concentrations of aplnergic peptides and mineral-bone peptides

**Figure 2:**
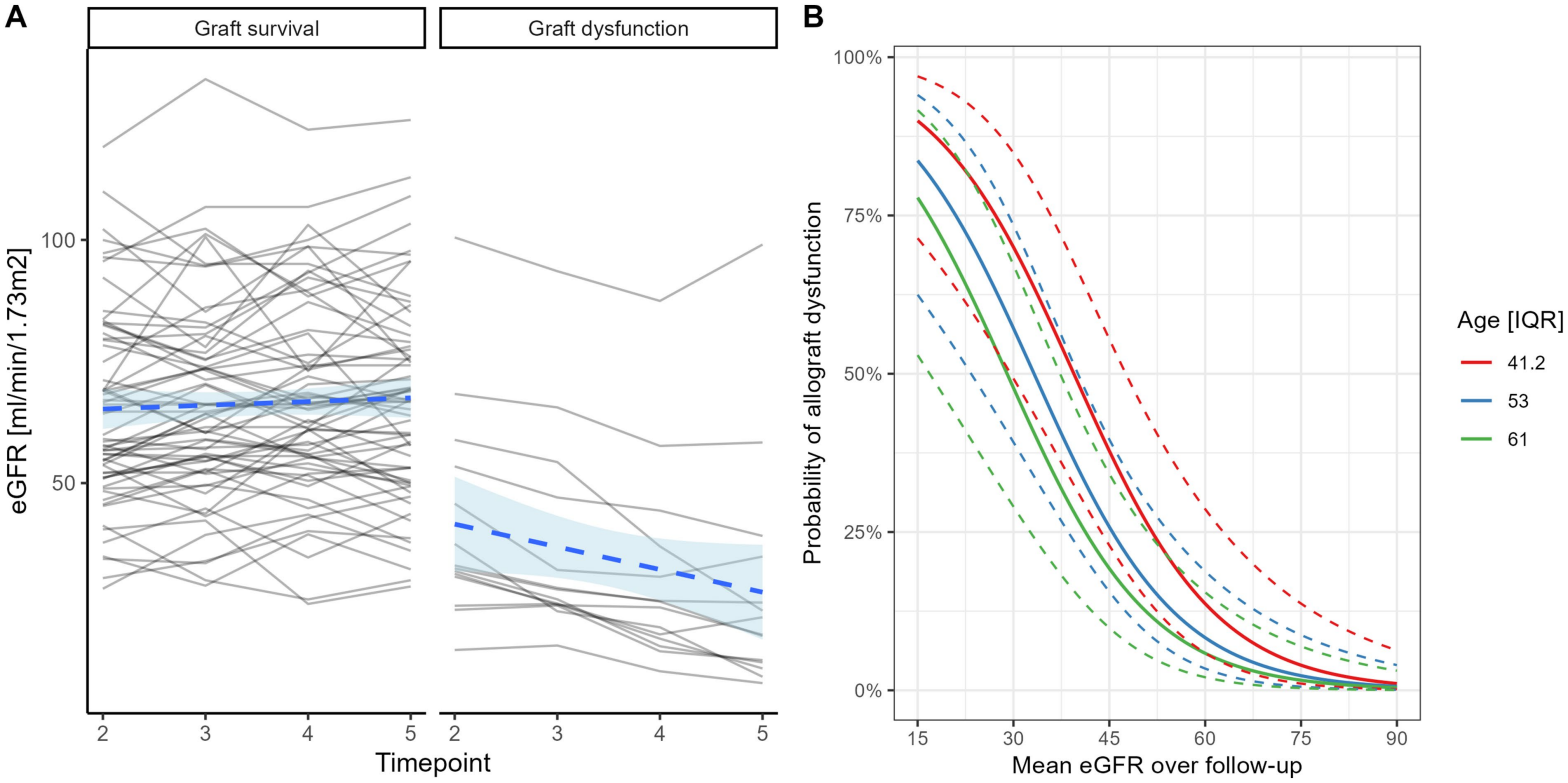
Temporal changes in renal function based on repeat eGFR and stratified by graft loss status **(A)**. Predicted probabilities of renal allograft loss based on mean eGFR values calculated over time and age group based on quartiles (logistic regression—**B**).

This cohort included patients from a broad range of kidney graft function at inception. When examining temporal changes in eGFR, we observed that patients who maintain allograft function have stable trajectories, as compared with subjects who (at a later date) required permanent dialysis transfer/transplantation re-listing and exhibited a gradual trend of eGFR decline (Figure 2A). In an age-adjusted logistic regression model (optimism corrected Somer’s Dxy 0.76, *R*^2^ = 0.51) for death-censored permanent dialysis requirement, higher mean eGFR over two-year follow-up was associated with significantly lower odds of allograft loss (OR 0.04, 95% CI 0.01–0.15; *p* < 0.001). The predicted probabilities for allograft dysfunction are illustrated and adjusted for interquartile age-range (Figure 2B).

Baseline renal function showed no significant association with aplnergic markers (*r* = −0.07, *p* = 0.51 for APLN, *r* = 0.17, *p* = 0.10 for ELA), in contrast to mineral bone peptides (*r* = −0.24, *p* = 0.02 for log(FGF-23); *r* = 0.34, *p* < 0.001 for log(α Klotho), respectively). Only a weak and inverse linear trend could be observed between serum APLN and ELA (*r* = −0.20, *p* = 0.04), as well as between log(FGF-23) and log(α Klotho) (*r* = −0.15, *p* = 0.14).

**Figure 3:**
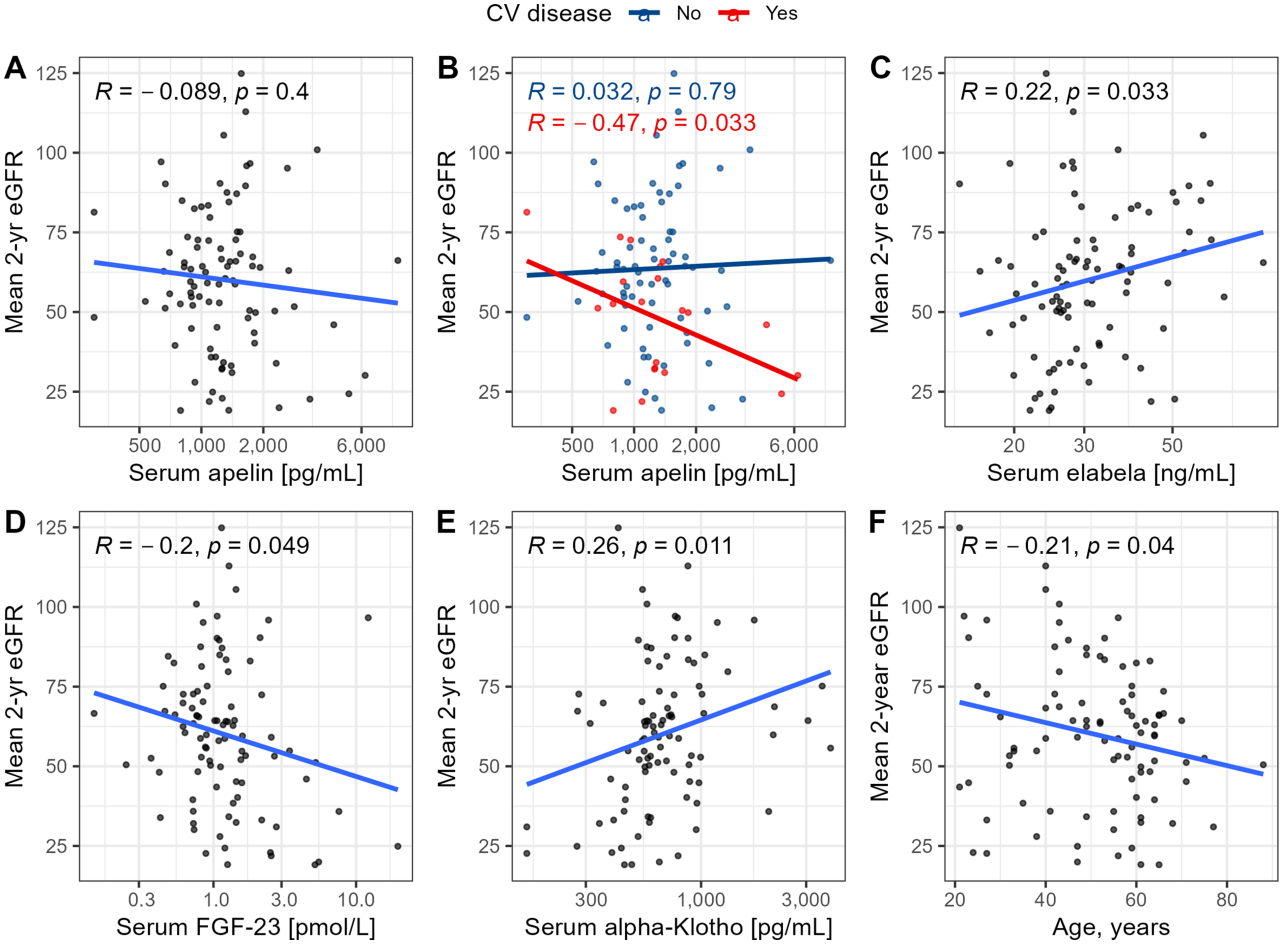
Relationships between mean eGFR over 2-year follow-up and baseline serum concentrations of APLN **(A, B)**, ELA **(C)**, FGF-23 **(D)**, alpha Klotho **(E)** and patients’ age **(F)**. Analyses were conducted after excluding patients with initial eGFR <30 ml/min/1.73m^2^.

To exclude the potential interference of impaired kidney clearance (KDIGO stage 4–5), we also explored relationships between circulating concentrations of aplnergic peptides and mineral-bone hormones with future eGFR over 2-year follow-up (Figure 3).

### 3.3 Prognostic models for future kidney function

**Figure 4:**
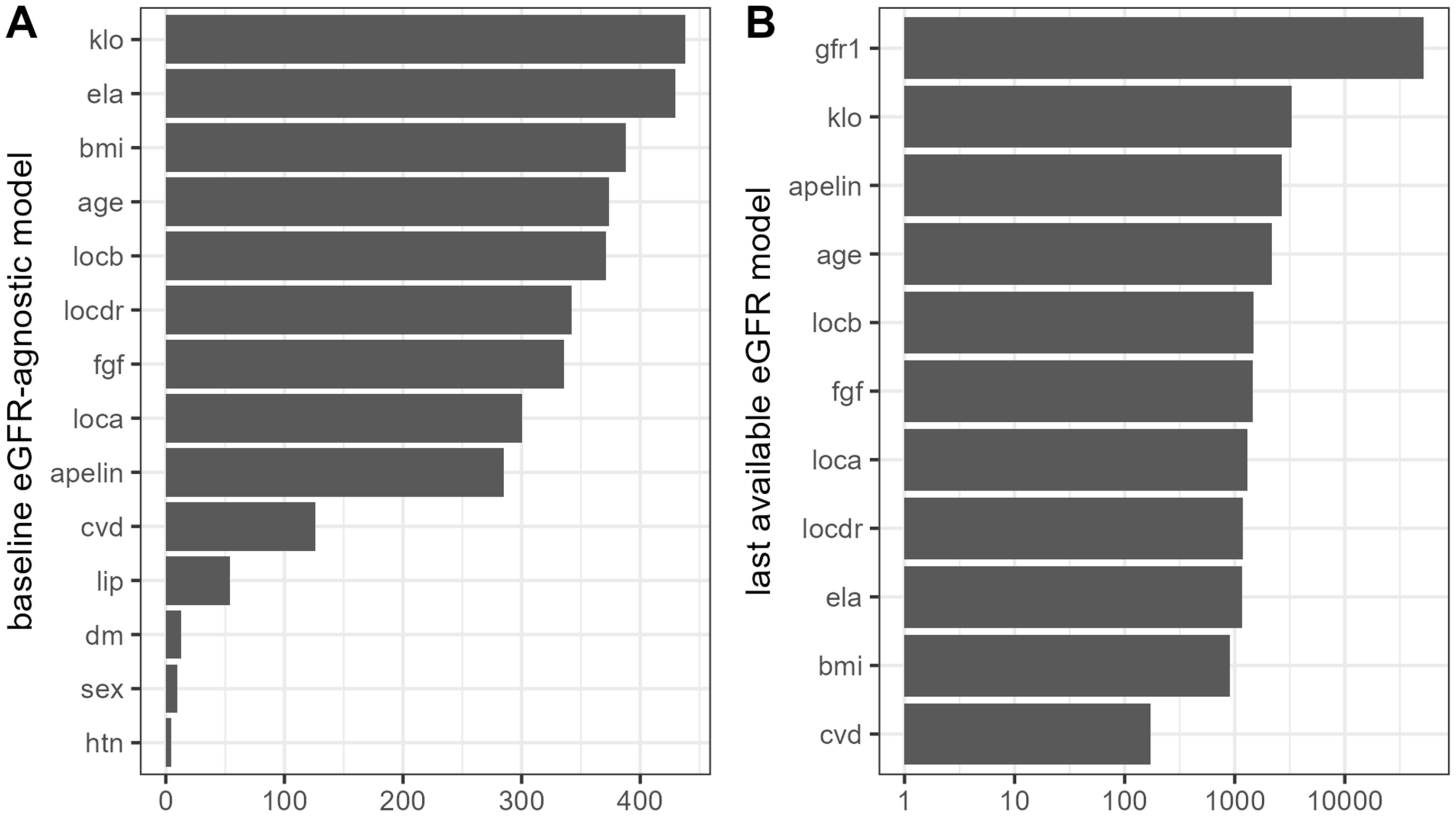
Variable importance for “full” models predicting mean future 2-year eGFR minus baseline eGFR **(A)** and last available eGFR **(B)** based on random forest models.

To understand the importance of baseline eGFR, a univariable RF regression model was trained and estimated to explain 87% of variance (R^2^ [SE]: 0.873 [0.010]) in future two-year eGFR. This suggests the overarching value of eGFR assessments over follow-up in stable KTRs. Therefore, to better evaluate the contribution of other clinical features, the mean eGFR over follow-up—baseline eGFR was adopted as a measure of kidney outcome. Constructing a model with all other available clinical characteristics, we estimated model performance with the ability to explain 13% of variance (R^2^ [SE]: 0.127 [0.023]) in future eGFR, agnostic of baseline eGFR. Based on global variable importance calculated for this model, we selected the 10 of the highest ranked features (α Klotho, ELA, BMI, Age, locus B mismatch, locus DR mismatch, FGF-23, locus A mismatch, APLN and CV disease) for further consideration. This trimmed model was validated respective to the last available eGFR over follow-up. Performance was estimated at 61% of variance (R^2^ [SE]: 0.613 [0.029]) in most recent eGFR. The change in the target outcome was predicated on selecting a separate final kidney measure that would least be correlated with initial eGFR (ie, in contrast to mean eGFR based on sequential measurements), but also with the iterative modeling process. A comparison of variable importance for highest ranked features between models is provided in Figure 4.

**Figure 5:**
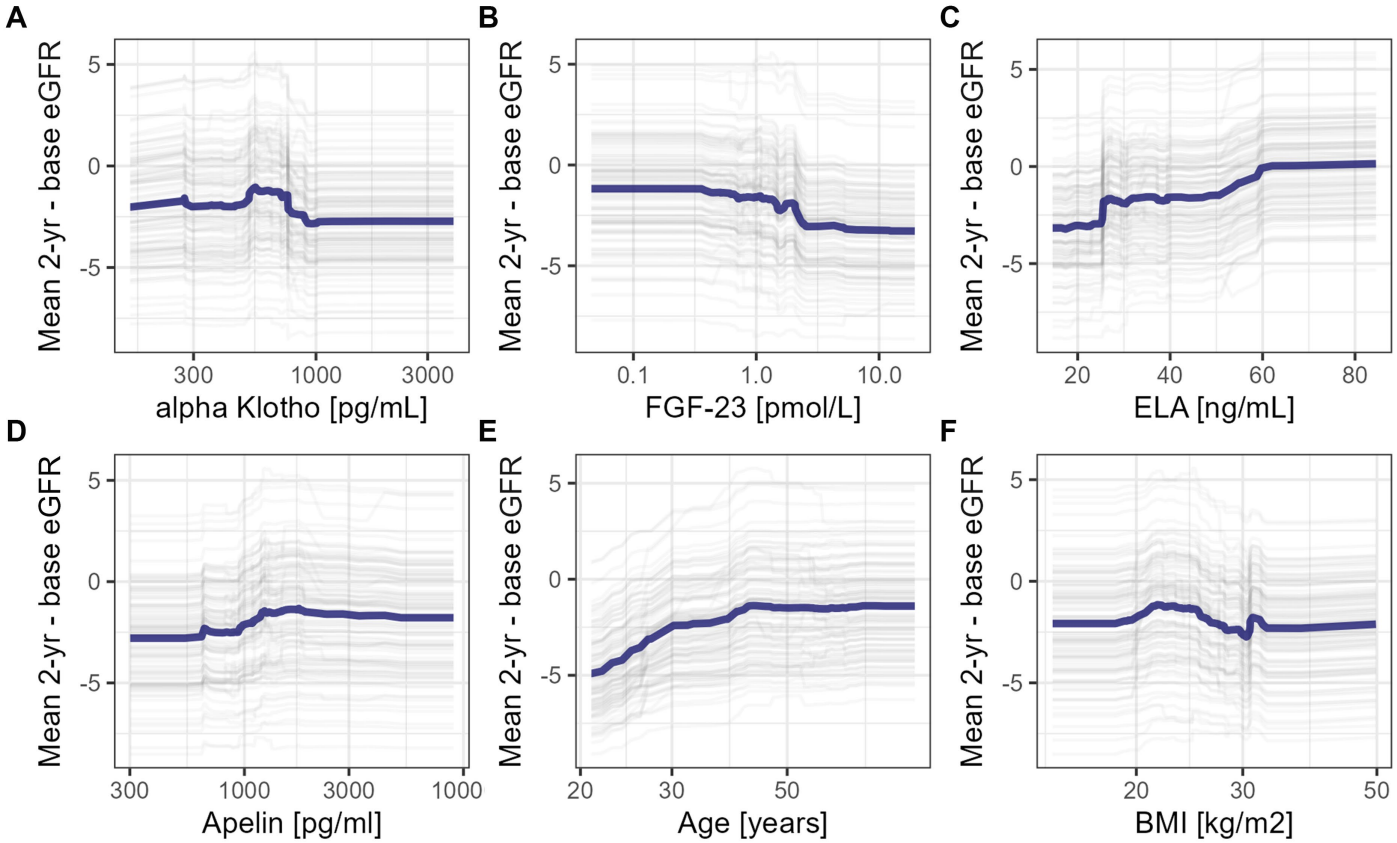
Partial dependence plots for marginal effects of selected clinical features: mean eGFR over 2-year follow-up and baseline serum concentrations of alpha Klotho **(A)**, FGF-23 **(B)**, ELA **(C)**, APLN **(D)**, patients’ age **(E)** and body-mass index (BMI) **(F)**.

Thereafter, we examined the marginal effects of candidate serum biomarkers based on the baseline eGFR independent model (Figure 5). Higher FGF-23 showed a negative relationship with deterioration in renal function, in contrast to aplnergic peptides. For BMI, the modeled relationship appeared non-monotonic and suggestive of better prognosis only in individuals with normal body mass. Patient age followed a log-like relationship, indicating a more favorable prognosis after the fourth decade.

For comparative purposes, we also constructed several univariable Cox Proportional Hazard regression models for each of the investigated candidate biomarkers. Changes in log(α Klotho) (HR 0.26 95% CI 0.12–0.58; *p* = 0.001) and log(FGF-23) (HR 2.14 1.49–3.09; *p* < 0.001) were significantly associated with death-censored allograft loss, in contrast to APLN (HR 1.00 95% CI 1.00–1.00; *p* = 0.383) and ELA concentrations (HR 0.98 95% CI 0.94–1.01; *p* = 0.209).

**Figure 6:**
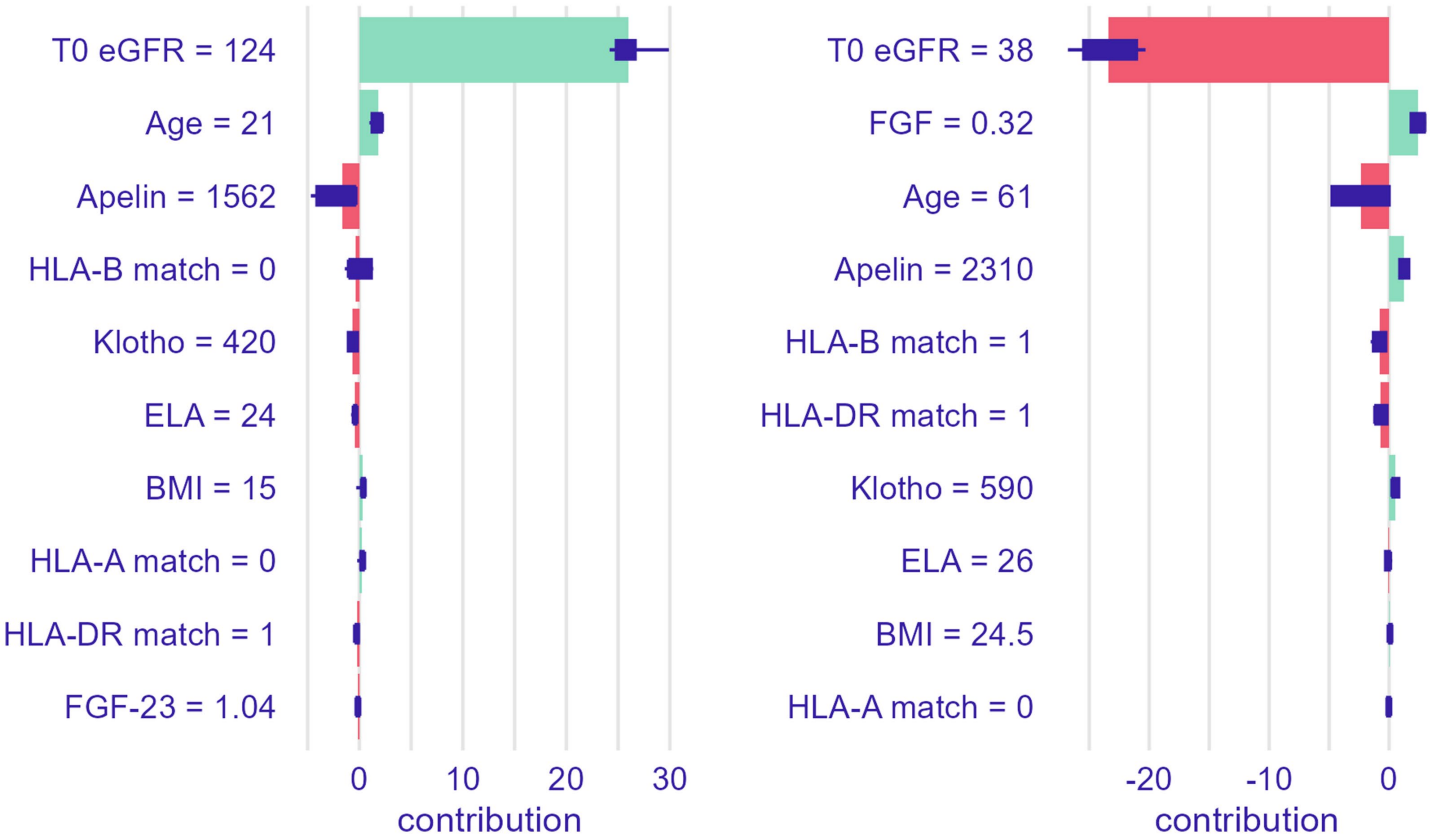
Local prediction breakdown for last available eGFR over follow-up based on two real-life patient cases.

Finally, to illustrate the prognostic contribution of selected clinical features, two patient cases were broken down based on the Shappley value approach (Figure 6). These local breakdown plots illustrate the overarching importance of initial eGFR in the late post-transplant period, but also the potential supplemental value of serum marker assessments, which can be integrated to improve predictions.

## 4 Discussion

Trends in survival of KTRs imply an improvement in early prognosis, which has been attributed to modern day treatment schemes (23–25). While acute rejection episodes and initial graft function remain a crucial determinant of early outcome (26), less is known about risk stratification in the late term. In this longitudinal cohort study of long-term KTRs, we observed a significant association between two-year renal function trajectories and circulating concentrations of both aplnergic and mineral-bone peptide markers. However, with the use of machine learning tools, we demonstrate that baseline eGFR remains the most salient predictor of future renal function. Incorporation of other clinical characteristics, or biochemical assessments of candidate serum markers, into multivariable models indicates their modest, but supplemental value in stratifying renal risk. The case of long-term KTRs is particularly noteworthy, as it reflects a setting of stable and sufficient immune de-sensitization, wherein other risk factors (i.e., for poor outcome) emerge in importance, such as the presence of CV disease and metabolic dysregulation (19, 27).

There are several theoretical considerations that justify investigation of the aplnergic system as a prognostic measure in KTRs. ELA and APLN show partially overlapping distribution throughout the vasculature (15, 28). Their joint receptor activation promotes vasodilation (15, 29) and increases cardiac contractility (15, 30), suggesting importance in a compensatory response to vascular disease. Meanwhile, within the kidney, APJ expression is more prevalent within glomeruli, indicating its hypothetical utility as a measure of glomerular, rather than tubular injury (16, 31, 32). Kidney injury results in APLN expression (33), while ELA mRNA levels are markedly reduced in ischemia–reperfusion models (34), suggesting an inverse role in the reparative response. Both aplnergic agonists are attributed cardio-(35, 36) and renoprotective (10, 37, 38) roles, though the activity of ELA is described as more potent (34). Thus, alterations in ELA, rather than APLN concentrations, are expected to parallel impairment within the kidney vasculature.

Among the investigated markers, significantly higher concentrations of ELA, FGF-23, APLN and lower α-Klotho concentrations were observed among KTRs, as compared with healthy controls. Both ELA and α-Klotho serum concentrations showed a significant relationship with future eGFR, while a similar association for APLN was noted only among individuals with overt CV disease. Using a robust machine-learning technique, we constructed predictive models to assist in understanding the prognostic contribution of these novel markers, which may be comparable to clinically established covariates. However, the overarching importance of eGFR at prior visits is also evident for patients with long-term, stable kidney allograft.

In patients with kidney disease, plasma APLN concentrations were first described by Malyszko et al. (39). Lower concentrations of APLN were tied to CVD, with ventricular dimensions in echocardiography as its major determinants, speculated to parallel the extent of hemodynamic overload (39). However, these were patients undergoing dialysis treatment, which is a unique setting. Another study in autosomal dominant polycystic kidney disease noted an independent association between APLN and renal function. The authors hypothesized it may parallel dehydration status and suggested the utility of circulating APLN as a prognostic measure, beyond the information derived from eGFR estimates (40). In patients with type 2 diabetes, serum APLN concentrations were observed in markedly higher concentrations, as compared with healthy subjects. Moreover, they were associated with albuminuria, an early indicator of diabetic nephropathy. Experimental evidence also indicates APLN may modulate the permeability and proliferation capacity of glomerular endothelial cells, which exerts downstream effects on hyperfiltration in glomeruli (41). In contrast, a report by Lu et al. showed no significant differences in APLN concentrations across patients with different stages of CKD when comparing with age- and sex-matched controls, though the authors did not account for CV status (42). Further work is necessary to clarify the relevance of circulating APLN concentrations.

Prior studies have suggested that ELA may be associated with progression of nephropathy (42). Data from two study groups suggests that aside from selective expression within the kidney, it is localized in tubular, rather than glomerular compartments (16, 43). ELA is a likely regulator of fluid homeostasis through its interaction with the Gi signaling pathway (17), though it may also act to antagonize aldosterone-stimulation of kidney tissue (44). Numerous experimental reports identify ELA expression as an effective countermeasure to renal injury (15, 43), though whether this is mechanistically linked to cell cycle and/or anti-inflammatory effects remains unclear (6).

Aplnergic activity is ascribed a protective role in arterial calcification processes, in part due to regulation of smooth muscle cell differentiation into osteoblast lines (45), but also attenuation of calcification processes (46). While mineral-bone peptides (FGF-23 and α-Klotho) did not show any apparent association with aplnergic activity, they shared an association with renal function over follow-up and contributed to the prediction of risk in multivariable models. We hypothesize that the pathogenic state of FGF-23 excess and α-Klotho deficiency slowly develops post-KTx, in a manner akin to progressing CKD, which also induces accelerated cellular aging processes (47). Therefore, the supplemental value of these peptide markers may parallel kidney and vascular dysfunction [similar to what has been observed in the general population (48)].

Traditional CV risk factors, with underlying metabolic disorders and excessive RAAS activation, are processes that promote allograft dysfunction (49). APLN shares a close relationship with TNF α expression in adipose tissue, which has led to its consideration as a potential bridge mediator between inflammation and insulin resistance in obesity (7, 50), though body-mass is likely not a major determinant of its circulating forms (51). Early-onset systemic inflammation post KTx has been demonstrated to be associated with long-term graft loss (52). In patients with type 2 diabetes, serum ELA levels are inversely tied to proteinuria and creatinine elevation (53), while experimental evidence suggests a protective role of ELA that reduces podocyte apoptosis (54). Antagonism between the RAAS and APJ axes has been previously described (55, 56) and a simplified schematic is provided for the reader in Supplementary Figure S1. Importantly, on a systemic level, aplnergic activity may only be marked under conditions of tissue stress (57, 58), as demonstrated by experimental studies of myocardial contractility in failing hearts (59, 60). This may explain, at least in part, the conflicting findings of patient-level studies.

In the model building process we utilized novel, candidate markers of recently discovered biologic processes that could shape cardiorenal disease, which is of high interest for KTRs. However, interpretation of circulating concentrations of aplnergic and mineral-bone peptides should be undertaken with caution due to difficulties in causal attribution (respective to their purported biologic roles based on experimental data) and incomplete understanding of these molecules. Many studies are limited by utilization of binary outcomes derived from repeated eGFR measures. Inter- and intra-individual variation in eGFR assessments, as well as timepoint number and temporal relation should be considered. To improve clinical interpretation, we defined the response variables as mean sequential eGFR and last available eGFR. For both outcomes, a strong association with death-censored allograft loss was verified. However, it should be noted that due the sample size, we had to rely on performance estimation based on training set cross-validation, which carries inherent bias. Additionally, we did not consider multivariable modeling of hard outcomes using time-to-event models due to the low event rate.

We assessed both APLN and ELA in serum using enzyme-linked immunosorbent assays, as has been performed by others. The potential variability in bioactivity and tissue affinity of specific molecular isoforms, as well as different sources of circulating peptides requires clarification and in-depth study. Interactions within signaling pathways tied to vascular disease (e.g., shear stress, endothelial dysfunction) or immune dysregulation may further occlude our interpretation of marker alterations. The results of the present study may further be influenced by sample heterogeneity derived from other, unknown confounders. Unfortunately, since our data were gathered from routine clinical practice in an outpatient setting, we did not possess consistent information to account for other, relevant characteristics (e.g., proteinuria, donor specific antibodies) (25, 61–63).

## 5 Conclusion

Discovering new biomarkers with prognostic potential and developing a risk model for renal function loss in KTRs holds high clinical importance, as no single parameter is sufficient to identify patients at greatest risk for allograft loss, and routine biochemical and clinical tests are often insufficient to predict graft failure accurately. The developing promise for aplnergic therapeutics in cardiac and renal diseases suggests another, supplemental role for APLN and ELA, as candidate biomarkers of a multisystem axis that counteract the effects of RAAS (1, 6, 10).

Following the early post-KTx stage, allograft loss is difficult to predict and complex models involving demographic, clinical and biochemical data remain of high interest. This was an exploratory, observational cohort study of long-term, stable KTRs, which examined temporal trajectories of renal function based on sequential eGFR assessments. Candidate serum markers tied to aplnergic activity and mineral-bone imbalance were evaluated, with theoretical justification derived from modulatory activity within vasculature and renal tissue. Circulating concentrations of serum ELA, APLN, FGF-23 and α-Klotho concentrations were significantly associated with mean eGFR over 2-year follow-up and provided some supplemental value in predicting future renal function in robust models.

## Data Availability

The original contributions presented in the study are included in the article/Supplementary material, further inquiries can be directed to the corresponding author.
