# Supplementary material for: Risk prediction of kidney function in long-term kidney transplant recipients
Source: Front Med (Lausanne). 2025 Mar 20;12:1469363. doi: 10.3389/fmed.2025.1469363 (PMC11965586; doi:10.3389/fmed.2025.1469363)
Supplement: Supplementary file 1 [file Image_1.pdf]

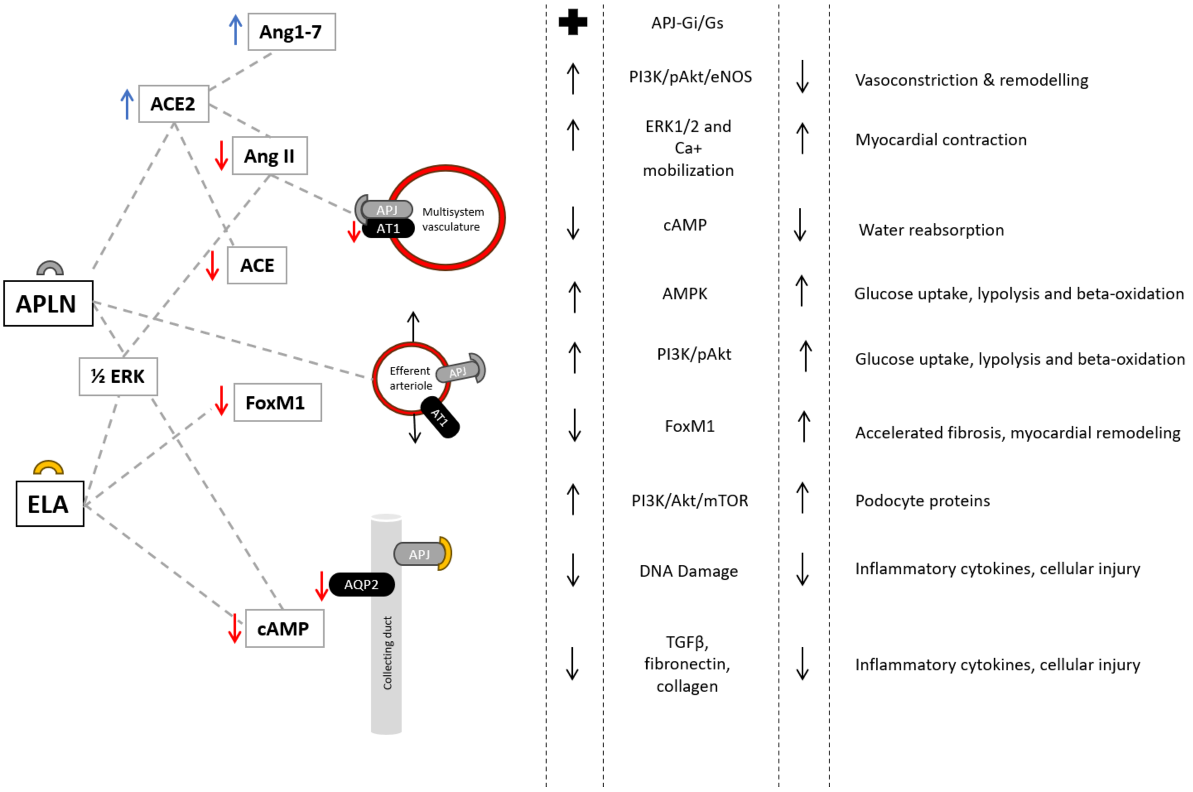

Supplementary Figure S1. Overview of pathomechanisms that may be involved in progression of nephropathy among kidney transplant recipients with associated biomarker hinge points.
